# Survey on request form content and result reporting in therapeutic drug monitoring service among laboratories in Czechia and Slovakia

**DOI:** 10.11613/BM.2020.020706

**Published:** 2020-06-15

**Authors:** Tomáš Šálek, Petr Schneiderka, Barbora Studená, Michaela Votroubková

**Affiliations:** 1 Department of Clinical Biochemistry and Pharmacology, Tomas Bata Hospital, Zlín, Czech Republic; 2 SEKK, spol. s.r.o., Pardubice, Czech Republic; 3 Institute of Clinical Biochemistry and Diagnostics, Medical Faculty in Hradec Králové, Charles University, Prague, Czech Republic; 4 Institute of Environmental and Chemical Engineering, University of Pardubice, Czech Republic

**Keywords:** drug monitoring, pharmacokinetics, preanalytical phase, clinical laboratory services, kidney function tests

## Abstract

**Introduction:**

The aim of the study was to investigate current practice and policies of therapeutic drug monitoring (TDM) service requesting and result reporting in Czechia and Slovakia.

**Materials and methods:**

All 149 laboratories that measure plasma drug concentrations were given an online questionnaire during a regular external quality assessment TDM cycle. The questionnaire consisted of 17 questions. The optimal TDM practice was defined as the application of all elements (age, body weight, time of sampling, date of the first administration, time of the last dose administration, the dose, the dosing interval, the route of administration, information on reason of testing, and information on other co–administered drugs) needed for reporting a recommendation for further drug dosing (positive response to question number 16).

**Results:**

The response rate was 69%, 103 out of 149 laboratories measuring drug concentrations. Only 12% (12 out of 103 laboratories) of the laboratories implemented all elements needed for optimal TDM practice and reported a recommendation. Both paper and electronic request forms were used by 77 out of 103 (75%) laboratories. A total of 69 out of 103 laboratories (67%) specified the type of sampling tube on their request form. Cystatin C was used for prediction of renal drug elimination by 24% (25 out of 103) of participants.

**Conclusions:**

Small number of laboratories implemented all elements needed for optimal TDM practice and report a recommendation on further dosing. Further efforts in education on optimal TDM practice as well as harmonization of service are desirable.

## Introduction

Therapeutic drug monitoring (TDM) is a part of laboratory medicine that enables correct drug dosing for medications with a narrow therapeutic index (*1*). Absorption, distribution, metabolism, and excretion are the most important pharmacokinetic variables. The clinical decision making on drug dosing cannot be performed without these variables. Consensus Guidelines for Therapeutic Drug Monitoring in Neuropsychopharmacology include exemplar request form with all elements needed for optimal TDM practice: age, body weight, time of sampling, date of the first administration, time of the last dose administration, the dose, the dosing interval, the route of administration, information on the reason of testing, information on other co–administered drugs. This guideline highly recommends that interpretation and pharmacologic advice are provided with every assessment of a drug concentration (*2*). The correct interpretation of plasma drug concentration is not possible without all of the required information on the exemplar request form in the Consensus Guidelines for Therapeutic Drug Monitoring in Neuropsychopharmacology. This TDM approach was demonstrated in patients overdosed with gentamicin (*3*). The practice is in accordance with a general trend in laboratory medicine that laboratory medicine is not limited only to the analytical phase but extra-analytical phases are equally important. All phases of a laboratory test should be harmonized. Interpretation of laboratory test results is a key part of the post-analytical phase (*4*). The integration of laboratory testing with pathology and imaging techniques is probably the future of laboratory medicine. This integration may lead to earlier and more accurate diagnosis (*5*).

Plasma drug concentrations are routinely measured by immunoassay methods that are prone to analytical interferences. Communication between laboratory and clinical specialists is needed in these cases (*6*). The measurement of plasma drug concentrations that are ordered by different clinical disciplines is centralized in medical laboratories. A questionnaire is an important tool to assess current laboratory practice in some fields of medicine (*7*). It may be also the first step in the development of guidelines (*8*).

The aim of the study is to investigate current practice and policies of TDM service requesting and result reporting in Czechia and Slovakia.

## Materials and methods

All 149 laboratories that measure plasma drug concentrations in Czechia and Slovakia could complete the electronic questionnaire that was added to routine external quality assessment (EQA) cycle of TDM from October 1^st^ to October 10^th^ 2019. The questionnaire was created by TDM cycle supervisors with the intent to cover the TDM topic overall. Another TDM supervisor validated the content by comparing it with the list of information that is needed to ensure good laboratory practice and interpretation of laboratory test results. The questionnaire was placed and distributed by an external quality assessment provider SEKK s. r. o. website www.sekk.cz to all 149 possible participants. The results of the drug concentration tests and answers to the questionnaire were entered electronically on SEKK’s EQA provider website.

All responses were taken into consideration and no answers were excluded. The questionnaire had an important educational role to educate laboratory professionals on the importance of pharmacokinetic parameters and their implications for drug dose adjustment. The list of the questions in the questionnaire is displayed in Table 1.

**Table 1:** Responses to the questionnaire by participating laboratories on their TDM practice

**Question number**	**Question text**	**Total number of answers**	**Answers (N)**
1	How many drugs does your laboratory measure?	103	1–5 drugs measured (71) 6–9 drugs measured (17) 10–56 drugs measured (15)
2	What type of a TDM request form do you use?	103	Only paper (22) Only electronic (4) Both paper and electronic (77)
3	Do you provide recommended type of tube for particular drug concentration measurement on your request form?	103	No (34) Yes (69)
4	What information do you require to fill on your request form?	102	Only requirement for drug concentration measurement (92) All pharmacokinetic parameter should be added (10) No answer (1)
5	Do you require information on time of sampling?	103	No (5) Yes (98)
6	Do you require information on time of the last dose administration?	102	No (89) Yes (13) No answer (1)
7	Do you require information on the dosing interval (such as 24 hours)?	102	No (94) Yes (8) No answer (1)
8	Do you require information on age?	102	No (3) Yes (99) No answer (1)
9	Do you require information on body weight?	102	No (78) Yes (24) No answer (1)
10	Do you require information on the dose?	102	No (92) Yes (10) No answer (1)
11	Do you require information on the route of administration?	103	No (95) Yes (8)
**Question number**	**Question text**	**Total number of answers**	**Answers (N)**
12	Do you require information on the reason of testing (such as signs of toxicity, suspicion of non-adherence to treatment)?	102	No (46) Yes (56) No answer (1)
13	Do you require information on other co–administered drugs and their dosing?	102	No (47) Yes (55) No answer (1)
14	Do you use any software for TDM modelling?	99	No (89) Yes (10) No answer (4)
15	What type of software do you use? (if applicable)	103	MWPharm (Mediware, Zuidhorn, the Netherlands) (10) We do not use any software (93)
16	Do you add an interpretative comment with dosing recommendation to plasma drug concentration? (if you have all pharmacokinetic data)	93	No (81) Yes (12) No answer (10)
17	What marker do you use for estimation of glomerular filtration rate in drugs excreted by kidneys?	94	Plasma creatinine (57) Plasma creatinine and cystatin C (25) Renal tests are not available (12) No answer (9)
TDM - Therapeutic drug monitoring.			

The optimal TDM practice was defined as the application of all elements (age, body weight, time of sampling, date of the first administration, time of the last dose administration, the dose, the dosing interval, the route of administration, information on the reason of testing, and information on other co–administered drugs) needed for reporting a recommendation for further drug dosing (positive response to the question number 16).

### Statistical analysis

Data were collected to Microsoft Word Office 2007 program (Microsoft, Washington, USA). The absolute and relative numbers of responses to particular questions were calculated by Microsoft Excel Office 2007 program (Microsoft, Washington, USA).

The denominator of all relative proportion calculations of answers to particular questions was the total number of participating laboratories (N = 103).

## Results

The response rate was 69% (103 out of 149 laboratories). Not all participants responded to all questions. Six questions were answered by all 103 participants of the survey. Other questions were not answered by all laboratories. Both paper and electronic request forms were used by 77 out of 103 (75%) laboratories.

Only 12% (12 out of 103 laboratories) of laboratories performed the optimal TDM practice with reporting an interpretative comment. These laboratories are university medical laboratories and laboratories of specialized centers such as a transplant hospital. They also require pharmacokinetic information on their request forms. They measure 10 or more drug concentrations. A total of 71 laboratories measured five or less drug concentrations. A total of 69 out of 103 laboratories (67%) specified the type of sampling tube on their request form. Cystatin C was used for prediction of renal drug elimination by 24% (25 out of 103) of participants. Responses to the questionnaire by participating laboratories are provided in Table 1.

## Discussion

We analysed the request form and reporting of results in therapeutic drug monitoring by medical laboratories in Czechia and Slovakia. Only 12% of laboratories adopted optimal TDM practice according to Consensus Guidelines for Therapeutic Drug Monitoring in Neuropsychopharmacology with request form including all elements needed for optimal TDM practice that enable reporting a recommendation for further drug dosing (positive response to the question number 16) (*2*). The majority of laboratories only measured drug concentrations.

Vecellio *et al.* found that manual data entry from hand written request forms into an electronic laboratory information system is prone to transcription errors (*9*). The majority of laboratories (75%) in this study had both paper and electronic request forms available, electronic request forms would prevent transcription error.

Dikmen *et al.* reported that serum separation gel can absorb some drugs and tubes with barrier gel are not allowed for TDM (*10*). Only 67% laboratories in the questionnaire study specified tube type on their request form.

Kang and Lee reported that trough plasma drug concentrations are most commonly used in routine TDM practice. The reason is that trough plasma concentrations are less influenced by absorption and distribution fluctuations compared to peak concentrations (*11*). The trough concentration is determined by the time of sampling before the next scheduled dose.

Al-Sulaiti reported the importance of peak drug concentration for vancomycin dosing. Peak-trough TDM approach improves the vancomycin-associated cure rate in patients treated with vancomycin for Gram-positive infections (*12*). The correct timing of blood draw is the major practical problem of the interpretation of peak plasma drug concentrations.

Yoon *et al*. showed that age, body-weight, and kidney function strongly affect serum vancomycin concentrations (*13*). It is the reason why these parameters should be included in the request form.

Kacirova *et al.* reported extreme metoprolol and propafenone serum concentrations in a patient with a lethal suicide attempt (*14*). Information on overdosing is very important for laboratory professionals because they can consider the analytical problem in situations of very high plasma drug concentration without this medical history.

Grundmann *et al.* found that TDM approach increased concentrations of lamotrigine due to interactions with valproic acid, despite lower doses of lamotrigine. Valproic acid decreased the clearance of lamotrigine by 66% in patients with this combination therapy (*15*). It shows the importance of knowledge of other drug administration when plasma drug concentration is found to be outside therapeutic ranges.

Barretto *et al.* reported in their review that two-thirds of all drugs are excreted by kidneys and cystatin C-based estimated glomerular filtration rate (eGFR) with cystatin C is probably superior compared to creatinine-based eGFR in the prediction of renal drug elimination (*16*). Only 24% (25 out of 103) of participants in our study used cystatin C for the prediction of renal drug elimination.

In his review article, Grubb reported a conclusion that cystatin C is indispensable for evaluation of kidney function (*17*). We try to promote its use but cystatin C is not widely used even in university labs probably due to higher cost compared to creatinine.

Grundmann *et al.* described TDM strategy with the collection of all pharmacokinetic information at his university center in patients treated with antipsychotic drugs (*18*). Our department also adopted this approach.

An Interpretative comment was included among quality indicators in the post-analytical phase (*19*). It may be desirable to include an interpretative comment with a recommendation regarding the next dose as a quality indicator of the post-analytical phase for medical laboratories.

Our study shows the current approach of medical laboratories in Czechia and Slovakia to TDM practice. There is a low number of interpretative comments in TDM reporting. The aim of this study is to improve the harmonization of TDM practice. The presence of an interpretative comment on the TDM report may be used as a quality indicator of the post-analytical phase.

Kacirova and Grundmann monitored monthly trough concentrations of gentamicin, amikacin, and vancomycin as an indicator of the quality of medical care and analysed the causes of potentially toxic concentrations (*20*). We also suggest that the rate of through drug concentrations above the therapeutic range may be used as a quality indicator.

The limitations of the study are incomplete response rate and our inability to verify responses. Some participants did not respond to all questions.

In summary, the small number of labs implemented all elements needed for optimal TDM practice and report a recommendation on further dosing. Harmonization of TDM service is desirable. The presence of an interpretative comment on the TDM report may be used as a quality indicator of the post-analytical phase.
